# GSK3-mediated raptor phosphorylation supports amino-acid-dependent mTORC1-directed signalling

**DOI:** 10.1042/BJ20150404

**Published:** 2015-08-20

**Authors:** Clare Stretton, Thorsten M. Hoffmann, Michael J. Munson, Alan Prescott, Peter M. Taylor, Ian G. Ganley, Harinder S. Hundal

**Affiliations:** * Division of Cell Signalling & Immunology, James Black Centre, College of Life Sciences, University of Dundee, Dundee DD1 5EH, U.K.; † MRC Protein Phosphorylation Unit, James Black Centre, College of Life Sciences, University of Dundee, Dundee DD1 5EH, U.K.

**Keywords:** amino acid, autophagy, growth, insulin, L-type (leucine) amino acid transporter 1 (LAT1), leucine, p70S6K1, proliferation, transcription factor EB (TFEB), sodium-coupled neutral amino acid transporter 2 (SNAT2), uncoordinated-51-like kinase (ULK1)

## Abstract

The mammalian or mechanistic target of rapamycin (mTOR) complex 1 (mTORC1) is a ubiquitously expressed multimeric protein kinase complex that integrates nutrient and growth factor signals for the co-ordinated regulation of cellular metabolism and cell growth. Herein, we demonstrate that suppressing the cellular activity of glycogen synthase kinase-3 (GSK3), by use of pharmacological inhibitors or shRNA-mediated gene silencing, results in substantial reduction in amino acid (AA)-regulated mTORC1-directed signalling, as assessed by phosphorylation of multiple downstream mTORC1 targets. We show that GSK3 regulates mTORC1 activity through its ability to phosphorylate the mTOR-associated scaffold protein raptor (regulatory-associated protein of mTOR) on Ser^859^. We further demonstrate that either GSK3 inhibition or expression of a S859A mutated raptor leads to reduced interaction between mTOR and raptor and under these circumstances, irrespective of AA availability, there is a consequential loss in phosphorylation of mTOR substrates, such as p70S6K1 (ribosomal S6 kinase 1) and uncoordinated-51-like kinase (ULK1), which results in increased autophagic flux and reduced cellular proliferation.

## Abstract (precis)

Glycogen synthase kinase-3 (GSK3) mediates phosphorylation of raptor on Ser^859^, which crucially supports activation of mechanistic target of rapamycin (mTOR) complex 1 (mTORC1) signalling in response to amino acid availability. GSK3 inhibition is associated with reduced mTORC1 signalling that impacts negatively on cell growth, protein synthesis and promotes cellular autophagy.

## INTRODUCTION

The mammalian or mechanistic target of rapamycin (mTOR) complex 1 (mTORC1) is a multimeric protein assembly composed of mTOR, mLST8 (mammalian lethal with SEC13 protein 8), proline-rich Akt substrate of 40 kDa (PRAS40) and raptor (regulatory-associated protein of mTOR) that integrates mitogenic and nutrient [amino acid (AA)] signals to regulate diverse cellular responses, including mRNA translation, cell growth/proliferation, metabolism and autophagy [1,2]. Activation of mTORC1 is crucially dependent upon a small G-protein called Rheb (Ras homolog enriched in brain), whose intrinsic GTPase activity is inhibited by the GTPase-activating protein (GAP) activity of the tuberous sclerosis complex (TSC1/2) [3]. TSC1/2 is itself subject to regulation by the IRS1–PI3K–Akt signalling axis whose activation by mitogenic stimuli results in Akt-mediated phosphorylation of TSC2 and inhibition of its GAP activity that, in turn, aids accumulation of active Rheb and a consequential increase in mTORC1 activity [4]. In contrast, stimulation of mTORC1 in response to nutrient availability occurs independently of the TSC1/2 complex [5] and is thought to involve translocation of mTORC1 from the cytosol to the lysosomal membrane, where mounting evidence suggests that sensing of AA sufficiency occurs. Members of the Rag family of small G-proteins have been implicated in the lysosomal recruitment of mTORC1 and operate as heterodimers (RagA–RagB and RagC–RagD) which promote redistribution of mTORC1 to lysosomal membranes in response to AA provision [6]. Rags are tethered to the lysosomal surface by interactions with two heteromeric protein complexes; the Ragulator (Rag regulator) complex [6] and the vacuolar H^+^-ATPase [7]. The Rag heterodimer is most active when the RagA–RagB component is GTP-loaded and the RagC–RagD component is in its GDP-bound form. In this state, the Rag heterodimer is able to bind mTORC1 allowing it to make contact with Rheb-GTP at the lysosomal surface where it becomes activated [7]. Once activated, the catalytic domain of mTOR phosphorylates substrates associated with raptor, which functions as a crucial scaffold placing bound substrates in close proximity to mTOR and enabling modulation of diverse downstream processes. Whereas the Ragulator possesses guanine-nucleotide-exchange factor (GEF) activity towards RagA/B to facilitate GDP exchange for GTP, hydrolysis of bound GTP is influenced by another protein complex, GTPase-activating protein towards Rags (GATOR1), which has GAP activity towards RagA–RagB [8]. Very recent work has now identified a lysosomal membrane AA transporter belonging to the solute carrier 38 (SLC38) family, SLC38A9 [also known as sodium-coupled neutral amino acid transporter 2 (SNAT9)], as a putative AA sensor whose occupancy by substrate AAs is thought to signal AA sufficiency to mTORC1 via its interaction with the Rag–Ragulator complex [9,10].

A further level of control of mTOR activity may be afforded by the stimulus (insulin and/or AA)-dependent regulation of TSC localization [11,12]. Insulin and AA provision have been shown to induce TSC dissociation from lysosomal membranes, whereas removal of either stimulus facilitates greater lysosomal association of the complex that will have a negative impact on Rheb activity, thereby providing a mechanism for repressing or ‘switching-off’ mTOR activity. However, despite the significant advances that have been made in our understanding of how mTORC1 is activated, certain aspects of the current model require further refinement in the light of recent studies demonstrating: (i) that AAs can induce mTORC1 activation without changing the guanyl nucleotide charging of Rag GTPases [13], and (ii) that leucine can promote mTORC1 activity independently of lysosomal mTOR localization [14]. Furthermore, given the diversity of signals that mTORC1 is able to integrate and the range of downstream cellular responses that it regulates, it is highly likely that other, hitherto unidentified, regulatory proteins are involved in controlling the fidelity with which activation and signalling of mTORC1 is achieved. Indeed, in addition to AA sensing and recruitment of the mTORC1 protein ensemble to the lysosomal membrane, the covalent modulation of key components of mTORC1 may serve as a crucial determinant of mTORC1 signalling. Previous work has highlighted, for example, that raptor undergoes multi-site phosphorylation upon catalytic activation of mTOR and that this may induce conformational changes in mTORC1 that permit phosphorylation of raptor-bound substrates by mTOR [15]. One of these sites on raptor, Ser^863^, is postulated to be phosphorylated by mTOR itself and is considered necessary for promoting the hierarchical phosphorylation of neighbouring residues (Ser^859^ and Ser^855^) by other, as yet, unidentified protein kinases [15]. Whilst raptor can be phosphorylated by RSK (p90 ribosomal S6 kinase) [16], AMPK (AMP-activated protein kinase) [17] and CDK1/cdc2 (cyclin dependent kinase 1) [18], there is no evidence to suggest that these kinases target these sites to support nutrient-dependent regulation of mTORC1. Another potential candidate kinase is glycogen synthase kinase 3 (GSK3). Recent evidence has highlighted that GSK3 positively regulates mTORC1 activity in MCF-7 breast cancer cells [19] and that it supports G_2_/M cell cycle progression through its ability to enhance mitotic raptor phosphorylation and mTORC1 activity [20].

In the present study, we show that raptor can be phosphorylated by GSK3 on Ser^859^ and that GSK3 plays a permissive role in AA-induced activation of mTORC1, given that pharmacological inhibition or silencing of GSK3 results in a striking reduction in AA-stimulated mTORC1 signalling. We also demonstrate that, although GSK3 inhibition/silencing has no detectable effect upon lysosomal mTOR localization or upon expression of key protein components of mTORC1, loss of Ser^859^ phosphorylation is associated with reduced interaction of raptor with mTOR resulting in a consequential reduction in the phosphorylation of downstream targets such as S6K1, 4E-BP1 (ukaryotic initiation factor 4E-binding protein) and ULK1 (uncoordinated-51-like kinase). This loss in mTORC1-mediated S6K1 and ULK1 phosphorylation is associated with reduced cell proliferation and increased cellular autophagy.

## MATERIALS AND METHODS

### Materials and reagents

Antibodies directed against GSK3α/β were purchased from Santa Cruz Biotechnology. Anti-GSKβ antibody was purchased from BD Biosciences. Anti-glyceraldehyde-3-phosphate dehydrogenase (GAPDH) and anti-FLAG antibodies were purchased from Sigma–Aldrich. Anti-LAMP2 antibody was from Abcam. Antibodies against GSK3α and raptor were obtained from the Division of Signal Transduction Therapy (DSTT), University of Dundee. Anti-light chain 3 (LC3) antibody was purchased from MBL. Anti-puromycin antibody was from Kerafast. Antibodies against raptor-Ser^863^ and raptor-Ser^859^ were kindly supplied by Dr Diane Fingar (University of Michigan, Ann Arbor, MI, U.S.A.). Alexa-Fluor 488-conjugated anti-mouse and anti-rabbit and Alexa-Fluor 594-conjugated anti-mouse secondary antibodies were from Life Technologies as was all cell culture medium. All other antibodies were purchased from Cell Signaling Technology. Rapamycin, insulin, SB415286 and SB216763 were purchased from Tocris Biosciences. CT99021 was a gift from Dr Calum Sutherland (University of Dundee). Roscovitine was from Merck. Bafilomycin A1 was from Enzo Life Sciences. [^32^P]ATP and Protein G–Sepharose were from GE Healthcare. Cycloheximide and puromycin were from Abcam. Ku-0063794 was a gift from Professor Doreen Cantrell (University of Dundee). Oligonucleotides were purchased from the University of Dundee Oligo Synthesis Service. All other reagents and chemicals were purchased from either Sigma–Aldrich or VWR, unless stated.

### Cell culture, gene-silencing and transfection

L6 rat skeletal muscle cells were grown as described previously in α-MEM containing 2% (v/v) FBS (Biosera) [21]. Experiments were carried out at the myotube stage, typically 7 days post-seeding. AA starvations were carried out by incubation in EBSS. AA resupply was carried out by incubation in EBSS containing a complete mix of AAs at plasma physiological concentrations. Other pharmacological inhibitors were added as described in the text and figure legends. Stable L6 GSK3α/β double knockdown cell lines were generated in a similar manner to that described previously using the oligonucleotides shown in Supplementary Table S1 [22,23]. Briefly, oligonucleotides were annealed as described previously and then cloned into the AgeI/EcoRI sites of either pLKO.1-puro (GSK3α hairpins only) or a pLKO.1-puro vector in which the puromycin-resistance gene had been removed and replaced with a sequence coding for hygromycin resistance (termed pLKO.1-hygro) at the *Bam*HI/*Kpn*I sites (GSK3β hairpins only). Generation of recombinant lentiviruses and transduction of L6 cell cultures were carried out as described previously [22] and stable cell lines were established by incubation with either 3 μg/ml puromycin or 15 μg/ml hygromycin. GSK3α knockdown cell lines were established first and then the GSK3β hairpins were transduced into these cell lines and re-selected. Control cell lines contain both the hygromycin- and the puromycin-resistant viruses containing a control non-targeting sequence made with the oligonucleotides shown in Supplementary Table S1. Stable cell lines were established using early passage cells and, once established, were only used for a maximum of five passages. Human embryonic kidney (HEK) 293T, U20S and HeLa cells were maintained in Dulbecco's modified Eagle's medium (DMEM) containing 10% FBS. HEK293T cells were transfected with pCMV–raptor wild-type (wt) and mutant constructs (8 μg of DNA/10 cm dish; 3 μg of DNA/six-well plate) using polyethyleneimine (PEI) with a 3:1 PEI/DNA ratio. Cells were used 48 h post-transfection. The effects of GSK3–mTORC1 inhibition upon the exponential growth phase of cells was monitored by seeding HEK293T cells at a seeding density of 3.5 × 10^4^ cells per well of a six-well dish. Cells were treated with vehicle control, SB415286 or rapamycin 24 h post seeding and then for periods up to 72 h with fresh medium and inhibitors being replaced every 24 h. Cells were trypsinized and counted in triplicate using a Neubauer counting chamber.

### Plasmids and mutagenesis

The vector pCMV–FLAG–raptor, containing human wt raptor sequence, was kindly provided by the DSTT (University of Dundee). Oligonucleotides shown in Supplementary Table S1 were used to create the S859A and S863A mutants using KOD Hot Start DNA polymerase (Novagen). The mutated raptor DNA regions were fully sequenced.

### Preparation of whole-cell lysates and immunoblotting

Cells were incubated as described in the text and figure legends. After treatment, cells were washed twice with ice-cold PBS and then lysed as described previously [21]. Protein concentrations in the final prepared lysates were determined using the Bradford method [24]. Cell lysates were separated using SDS/PAGE, transferred onto PVDF (Millipore) membranes and then immunoblotted with the appropriate primary antibodies followed by incubation with appropriate horseradish peroxidase (HRP)-conjugated anti-IgG antibody. Proteins were detected using ECL using exposure to autoradiographic film (Kodak). Quantification of immunoblots was carried out with ImageJ (NIH).

### GSK3 kinase activity assays

L6 cells were treated as described in the text and cells extracted from 10 cm dishes using lysis buffer [21]. GSK3 was immunoprecipitated from 100 μg of cell lysate for 2 h using Protein G–Sepharose beads that had previously been conjugated to isoform-specific antibodies for GSK3α and GSK3β. Antibody–bead complexes were then washed twice in lysis buffer containing an additional 0.5 M sodium chloride and twice in wash buffer (50 mM Tris/HCl, pH 7.5, 0.1 mM EGTA and 0.1% β-mercaptoethanol). Beads were resuspended in a final volume of 50 μl of assay buffer {50 mM Tris/HCl, pH 7.5, 0.1 mM EGTA, 0.1% β-mercaptoethanol, 50 mM magnesium acetate, 0.2 mM phospho-GS peptide (YRAAVPPSPSLSRHSSPHQSEDEEE), 100 μM non-radiolabelled ATP and 2.7 μCi of [^32^P]ATP} and incubated shaking for 30 min at 30°C. Thirty microlitres of the reaction mix was spotted on to P81 filter paper. Papers were washed three times in 1% phosphoric acid then once in acetone before being dried and [^32^P]ATP incorporation was measured using a Beckman LS 6000IC scintillation counter.

### RNA extraction, cDNA synthesis and real-time PCR

RNA was extracted from cells using TriReagent (Sigma–Aldrich) according to the manufacturer's instructions. First-strand cDNA was synthesized from 1 μg of total RNA using oligo(dT)_15_ primers and Moloney murine leukaemia virus reverse transcriptase (Promega) according to the manufacturer's instructions. Real-time quantitative PCR was performed using a StepOne Plus real-time thermocycler (Applied Biosystems), SYBR Green JumpStart kit (Sigma–Aldrich) and primers targeting GSK3α, GSK3β, L-type (leucine) amino acid transporter 1 (LAT1), SNAT2 and GAPDH as a control. Sequences of primers used are shown in Supplementary Table S1. PCR conditions were as follows: initial denaturation at 95°C for 2 min followed by 40 cycles of 95°C for 15 s, 55°C for 15 s and extension at 68°C for 30 s. The ratio of GSK3, LAT1 and SNAT2 expression to *GAPDH* mRNA expression was calculated using a method described previously [25].

### Quantification of LC3 puncta and TFEB immunofluorescence

U2OS cells were seeded out on to glass coverslips. Twenty-four hours later, cells were treated with AA/inhibitors as indicated in the figure legends, except in the final 15 min when cells were incubated in the absence or presence of 100 nM bafilomycin A1 (Enzo Life Sciences). Cells were subsequently fixed in 3.7% formaldehyde, permeabilized with 0.2% NP-40 and stained with mouse anti-LC3 (1:1000, MBL International Corporation) followed by Alexa Fluor 488-conjugated anti-mouse secondary antibody (Life Technologies). Slides were stained and mounted using ProLong Gold antifade reagent with DAPI (Life Technologies) to enable localization of nuclei and viewed on a Nikon Eclipse Ti widefield microscope and quantified from three fields of view (with a minimum of 25 cells per field) per condition utilizing NIS-Elements software. For TFEB (transcription factor EB) localization studies, HeLa cells were seeded on to glass coverslips. At approximately 70% confluency, cells were transfected with 2 μg of the plasmid pcDNA5-FRT/TO-GFP TFEB wt (a gift from the laboratory of Professor Carol MacKintosh, University of Dundee) using the Metafectene+transfection reagent (Biontex). Twenty-four hours later, cells were treated as described in the text and figure legends, fixed in 4% paraformaldehyde for 10 min, then mounted in Vectashield DAPI-containing mounting medium (Vector Laboratories). For mTOR localization studies, HeLa cells were seeded on to coverslips and grown until approximately 70% confluent. Treatments were carried out as described in the text and figure legends. Cells were fixed in 4% paraformaldehyde for 10 min then permeabilized for 10 min with 1% Triton X-100. Blocking was carried out for 1 h at room temperature (RT) in 10% goat serum/0.2% BSA/PBS then primary antibodies were incubated on the coverslips overnight at 4°C in a humidified chamber. Following washing, the appropriate secondary antibodies were incubated on the coverslips for 1 h at RT. Coverslips were washed and mounted in VectaShield DAPI-containing mounting medium. Cells were imaged on a Zeiss LSM 700 confocal microscope and images were quantified using Volocity software (PerkinElmer) version 6.3.0. Briefly, DAPI-stained nuclei and GFP-transfected cells were identified using Otsu's method, nuclei areas were subtracted from the identified cells to give a cytoplasmic GFP intensity and this was used to give a nuclear/cytoplasmic intensity ratio for each image. Ten images were taken for each treatment.

### Protein synthesis

Protein synthesis was measured as described by Kelleher et al. [26] by assaying the incorporation of puromycin into newly synthesized peptides. Briefly, cells were pre-treated as described in the figure legends with AAs, insulin or cycloheximide (50 μg/ml) prior to incubation in the absence or presence of 1 μM puromycin for 30 min. At the end of this period, cells were lysed and lysates were subjected to SDS/PAGE and immunoblotting of PVDF membranes carried out overnight at 4°C with a mouse monoclonal anti-puromycin antibody [1 μg/ml in Tris-buffered saline with 0.01% (v/v) Tween 20 and 5% (w/v) non-fat dried milk] followed by incubation with goat anti-mouse HRP-conjugated secondary antibody.

### LC–MS/MS

HEK293T cells were treated with or without SB415286 for 1 h and lysed with lysis buffer containing 50 mM HEPES, pH 7.4, 150 mM NaCl, 1 mM EDTA, 10% (v/v) glycerol, 0.5% (v/v) NP-40, 1 mM DTT, 1 mM PMSF and phosphatase inhibitors. Lysates were clarified by centrifugation at 21000 ***g*** for 10 min at 4°C. Raptor was directly immunoprecipitated using an antibody raised against human raptor (residues 1–20). Samples were resolved by SDS/PAGE, and acrylamide gels were subsequently stained for protein using Instant Blue™ Coomasssie Blue (Expedeon) as per the manufacturer's guidelines. Bands corresponding to raptor were excised and diced into small cubes (∼1 mm) and transferred to a clean Eppendorf per band.

Gel pieces underwent sequential washes (0.5 ml for 10 min each on a vibrating platform) with water, 50% acetonitrile (ACN), 100 mM ammonium bicarbonate (NH_4_HCO_3_) and 50% ACN/50 mM NH_4_HCO_3_. Samples were alkylated in-gel; first samples were reduced by addition of 75 μl of 10 mM DTT in 0.1 M NH_4_HCO_3_ for 45 min at 65°C. The supernatant was removed and then 75 μl of 50 mM iodoacetamide in 0.1 M NH_4_HCO_3_ was used to alkylate samples for 20 min at RT. Supernatant was removed and gel pieces were washed with 50 mM NH_4_HCO_3_+50% ACN. Gel pieces were incubated with 0.3 ml of ACN for 15 min at RT; this was removed by centrifugal evaporation (SpeedVac™, Thermo Scientific) to dry the gel pieces. To digest proteins, 30 μl of 25 mM triethylammonium bicarbonate containing 5 μg/ml trypsin was added to gel pieces and incubated shaking for 30 min at RT. Water was added, if required, to completely cover gel pieces, and samples were left shaking at 1100 rev/min for 16 h at RT in a Thermomixer® (Eppendorf). An equal volume of ACN was then added to the gel pieces to extract peptides and incubated at 1100 rev/min for 15 min at RT. The supernatant was removed and dried by centrifugal evaporation. During this time, 100 μl of 50% ACN and 2.5% formic acid was added to the gel pieces and shaken at 1100 rev/min at RT. The supernatant was added to the initial dried extract and dried once more by centrifugal evaporation. Once completed samples were submitted to the MRC proteomics and MS team for analysis by LC–MS/MS.

### mTOR and raptor immunoprecipitation

HEK293T cells were cultured in 10 cm plates and treated as described in figure legends. Cells were washed twice in ice-cold PBS and lysed in HEPES/CHAPS buffer (40 mM HEPES, pH 7.4, 120 mM NaCl, 1 mM EDTA and 0.3% CHAPS) supplemented with 1 mM DTT, 1 mM PMSF and phosphatase inhibitors. Endogenous mTOR was immunoprecipitated from 2 mg of lysate using 10 μg of anti-mTOR antibody (obtained from the DSTT, University of Dundee) and 25 μl of Protein G–Sepharose. Beads were washed with HEPES/CHAPS lysis buffer, and proteins were eluted and analysed by Western blotting. Alternatively, for immunoprecipitation of FLAG–raptor, HEK293T cells expressing wt or mutant FLAG–raptor were treated and lysed as described above. FLAG–raptor variants were immunoprecipitated from 1 mg of lysate by incubation with 4 μg of anti-FLAG antibody (Sigma) for 2 h. After 1 h of incubation, 30 μl of Protein G–Sepharose beads were added. Beads were washed twice with HEPES/CHAPS lysis buffer and 380 mM NaCl and twice with HEPES/CHAPS lysis buffer before resuspending in SDS sample buffer.

### Analysis of GSK3 activity and raptor-associated mTORC1 kinase activity

L6 cells were treated as described in the figure legends and cells were extracted from 10 cm dishes using lysis buffer [21]. GSK3 was immunoprecipitated from cell lysates, and isoform-specific GSK3 activity assayed as described above. For analysis of raptor-associated mTOR kinase activity, cells were treated and lysed with HEPES/CHAPS buffer as described above. FLAG-tagged raptor was immunoprecipitated from 2 mg of lysate using anti-FLAG antibodies and 25 μl of Protein G–Sepharose as described above. Beads were washed with HEPES/CHAPS lysis buffer and subsequently resuspended in 30 μl of kinase assay buffer (25 mM HEPES, 50 mM KCl, 10 mM MnCl_2_, 200 μM ATP and 1 mM DTT). Kinase reactions were carried out for 10 min at 30°C in the presence of 3 μg of recombinant kinase-dead p70S6K (DSTT, University of Dundee). Reactions were terminated with 10 μl of 4× SDS sample buffer and p70S6K phosphorylation was visualized by SDS/PAGE and subsequent immunoblotting using an anti-p70S6K Thr^389^ antibody.

### *In vitro* raptor phosphorylation

Mutation of pEBG6/raptor (DSTT, University of Dundee) was carried out at the indicated sites using site-directed mutagenesis to generate triple alanine mutants of Ser^855/859/863^ and Ser^877/881/885^. HEK293T cells were cultured in 10 cm plates and 5 μg of plasmid (both wt and mutant) transfected using 25 μg of PEI per plate as a transfection reagent. The following day, cells were lysed as described above. GST-tagged raptor was immunoprecipitated in duplicate from 1 mg of lysate for 2 h using 20 μl of glutathione–Sepharose 4 Fast Flow (GE Healthcare). Protein–bead complexes were washed twice in lysis buffer containing an additional 0.5 M sodium chloride and twice in wash buffer (50 mM Tris/HCl, pH 7.5, 0.1 mM EGTA and 0.1% β-mercaptoethanol). Beads were resuspended in a final volume of 50 μl of assay buffer {50 mM Tris/HCl, pH 7.5, 0.1 mM EGTA and 0.1% β-mercaptoethanol, 50 mM magnesium acetate, recombinant GSK3β (DSTT, University of Dundee), 100 μM non-radiolabelled ATP and 2.7 μCi of [^32^P]ATP} and incubated on a shaker for 30 min at 30°C. Reactions were boiled with 10 μl of 6× Laemmli buffer for 15 min and 30 μl of separated by SDS/PAGE and transferred on to PVDF membrane, as described above. Incorporation of [^32^P]ATP was detected using exposure to radiographic film. Following this, membranes were blocked and probed with anti-GST antibody to confirm equal protein loading.

### Statistical analysis

Statistical analysis was performed using GraphPad Prism. Data are presented as means ± S.E.M. with quantitative graphics and statistical assessments being derived from a minimum of three biological replicates per group. Values used to normalize results in each assay were excluded from statistical analysis, which involved ANOVA and Tukey's post-test for multiple comparisons. Significance was set at a *P*-value of <0.05.

## RESULTS

### Pharmacological inhibition of GSK3 suppresses mTORC1 signalling

**Figure 1:**
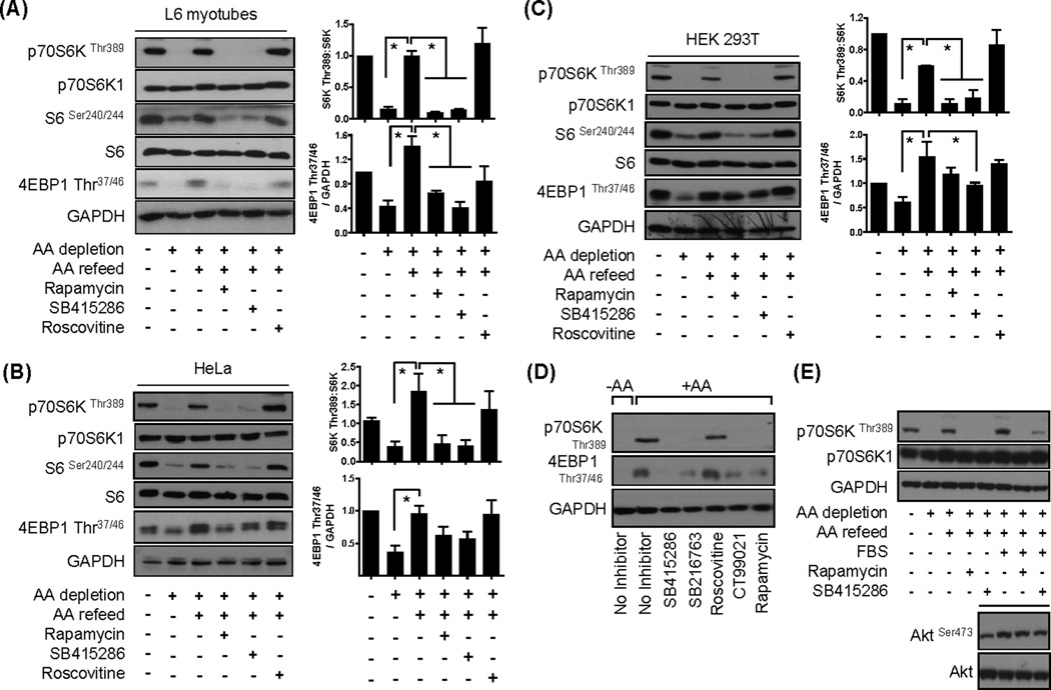
Effect of GSK3 inhibitors on mTORC1 signalling in L6 myotubes, HeLa and HEK293T cells (**A**–**C**) Cells were held in EBSS containing or lacking AAs for 1 h or alternatively having been depleted of AAs incubated in EBSS containing a 1× AA mix (refeed) for 15 min. Cells were incubated with 100 nM rapamycin, 50 μM SB415286 or 30 μM roscovitine for 15 min prior to and for 15 min during the AA-refeed period. Cells were lysed and 30 μg of lysate protein was analysed by immunoblotting. Blots shown in (**A**–**C**) are representative of a minimum of three independent experiments and histograms (means ± S.E.M., *P<0.05) show quantification of these. (**D**) L6 myotubes were AA-depleted for 1 h in EBSS followed by incubation in EBSS+AA for 15 min in the absence or presence of 50 μM SB415286, 10 μM SB216763, 30 μM roscovitine, 40 μM CT99021 or 100 nM rapamycin for 0.5 h. Cells were harvested and 30 μg of protein was analysed by immunoblotting using the antibodies indicated. (**E**) HEK293T cells were subjected to AA-depletion/refeeding as in (**A**–**C**) or incubated with EBSS supplemented with serum [10% (v/v) FBS] ± 100 nM rapamycin or 50 μM SB415286 for 0.5 h, as indicated. Cells were lysed and 30 μg of protein was analysed by immunoblotting using the antibodies indicated.

mTORC1-directed signalling is critically dependent upon AA availability and, as shown in Supplementary Figures S1(A) and S1(B), can be rapidly down- or up-regulated in cells irrespective of their lineage when they are subjected to withdrawal or resupply of extracellular AAs respectively. In an attempt to study whether GSK3 participates in the regulation of mTORC1 signalling, we initially investigated the effect of GSK3 inhibition upon AA-dependent phosphorylation of downstream mTORC1 substrates. Consistent with the findings shown in Supplementary Figure S1, the data presented in Figures 1(A)–1(C) indicate that subjecting L6 myotubes, HEK293T and HeLa cells to a 60-min period of AA depletion promotes a significant reduction in phosphorylation of p70S6K1^Thr389^ and 4E-BP1^Thr37/46^, whereas subsequently refeeding cells with a physiological 1× AA mix re-instates phosphorylation of both proteins to a level comparable to that seen in AA-replete cells. The phosphorylation status of ribosomal S6 is respectively down- and up-regulated when cells were either AA-deprived or-refed following AA depletion. The ability of AAs to promote p70S6K1^Thr389^ and S6^Ser240/244^ phosphorylation in all three cell lines was severely blunted by the mTOR inhibitor rapamycin (Figures 1A–1C). Although the AA-induced phosphorylation of 4E-BP1^Thr37/46^ was also blunted by rapamycin, the effect of the inhibitor on 4EBP-1 phosphorylation was not as potent as that on p70S6K1^Thr389^ phosphorylation in HeLa or HEK293T cells (Figures 1B and 1C). This finding is consistent with previous reports indicating that mTORC1 substrates, such as p70S6K1 and 4E-BP1, exhibit differential sensitivity towards rapamycin [27]. Strikingly, SB415286, a potent and selective cell-permeant, ATP-competitive inhibitor of GSK3α and GSK3β [28,29], also caused strong repression of the AA-induced phosphorylation of p70S6K1 and S6. Again, whereas AA-induced phosphorylation of 4E-BP1^Thr37/46^ was sensitive to SB415286, the observed inhibition was modest in comparison with that seen for p70S6K1 and S6. Since SB415286 has been shown to also inhibit members of the CDK family in *in vitro-*based kinase assays [30] and CDK1/cdc2 may modulate raptor phosphorylation [18], we also assessed the effects of roscovitine, a potent cdc2, CDK2 and CDK5 inhibitor [31], which does not inhibit GSK3 (Supplementary Figure S2A). Figures 1(A–C) show that, unlike rapamycin or SB415286, roscovitine did not attenuate AA-induced phosphorylation of mTORC1/S6K1 targets. The efficacy of roscovitine as a CDK inhibitor was confirmed in separate experiments demonstrating a reduction in immunoprecipitable CDK5 activity (results not shown). We also assessed the effect of two additional, but structurally distinct, GSK3 inhibitors, SB216763 and CT99021 [30]. Figure 1(D) shows that these GSK3 inhibitors also exerted a repressive effect on AA-induced mTORC1 signalling in L6 myotubes, thus strongly implicating GSK3 in the regulation of mTORC1 activity.

Although AA provision alone is an important stimulus for mTORC1, it is well recognized that activation of the complex is further enhanced by the presence of serum growth factors. Figure 1(E) shows that AA-induced phosphorylation of p70S6K1^Thr389^ by mTORC1 was augmented in HEK293T cells exposed to serum, but that this enhanced stimulation remains sensitive to SB415286. In contrast, it is noteworthy that SB415286 did not suppress serum-induced phosphorylation of the Ser^473^ site on Akt, which serves as a target for mTORC2 (Figure 1E, inset). It is important to stress that HEK293T cells exhibit elevated Akt activity in the basal (unstimulated) state [32] and for this reason any enhancement in Akt or p70S6K1 phosphorylation induced by serum treatment of these cells appears modest (Figure 1E).

### Muted mTORC1 signalling in GSK3-silenced cells

**Figure 2:**
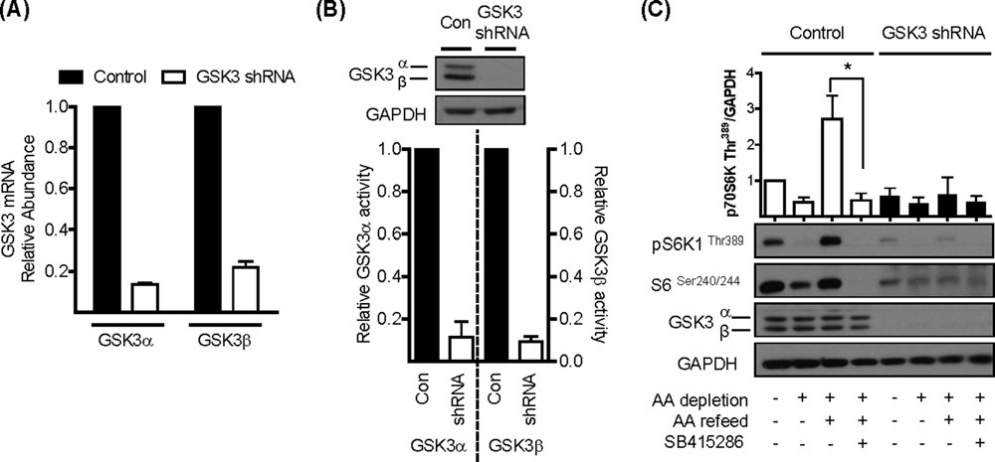
Effects of shRNA-mediated gene silencing of GSK3α/β upon mTORC1-directed signalling (**A**) Total RNA was extracted from L6 myotubes stably expressing either a non-specific shRNA or shRNAs targeting GSK3α and GSK3β. cDNA was synthesized from the mRNA and relative *GSK3* mRNA abundance, as measured against *GAPDH* mRNA, assessed by quantitative PCR. (**B**) Lysates were prepared from L6 myotubes stably expressing either a non-specific shRNA or shRNAs targeting GSK3α and GSK3β. A 30 μg amount of lysate was analysed by immunoblotting using anti-GSK3α/β or anti-GAPDH antibodies. In addition, 100 μg of lysate was also subjected to immunoprecipitation using antibodies against either GSK3α or GSK3β and analysis of the respective GSK3 activities carried out using a phospho-GS peptide as a substrate. (**C**) L6 myotubes stably expressing either a non-specific shRNA or shRNAs targeting GSK3α and GSK3β were held in EBSS containing or lacking AA for 1 h or alternatively having been depleted of AAs incubated in EBSS containing a 1× physiological AA mix (refeed) for 15 min in the absence or presence of 50 μM SB415286. Cells were harvested and 30 μg of lysate was analysed by immunoblotting using the antibodies indicated. The asterisk indicates a significant (*P*<0.05) change between the indicated bars.

To further substantiate the involvement of GSK3 in mTORC1 signalling, we utilized lentivirus-based shRNA technology to generate L6 myotubes exhibiting stable knockdown of both GSK3α and GSK3β. Figure 2(A) shows that the relative mRNA abundance for each GSK3 isoform was reduced by up to ∼85% by GSK3 shRNA, which resulted in substantial (∼90%) depletion of GSK3α and GSKβ protein and immunoprecipitable isoform-specific GSK3 activity (Figure 2B). Analysis of p70S6K1^Thr389^ and S6^Ser240/244^ phosphorylation revealed that, although cells expressing the control shRNAs exhibit the classical inhibitory and stimulatory response to AA withdrawal and resupply respectively, these responses were significantly reduced in shRNA GSK3-silenced cells (Figure 2C), suggesting that GSK3 activity is required to support mTORC1 signalling.

It is plausible that the reduced activation of mTORC1 signalling that we observe in GSK3 inhibitor-treated cells or those in which we have stably silenced GSK3α/β expression may be a consequence of reduced uptake of extracellular AAs. However, analysis of AA uptake and expression of LAT1 (SLC7A5) and the SNAT2 (SLC38A2) transporter reveals that for the latter there was no change in transport activity as a result of silencing GSK3, whereas uptake and accumulation of leucine (a potent activator of mTORC signalling [33]) by the former was actually elevated under conditions when GSK3 activity is inhibited (Supplementary Figure S3).

### Effects of GSK3 inhibition upon cellular autophagy, protein synthesis and cell proliferation

**Figure 3:**
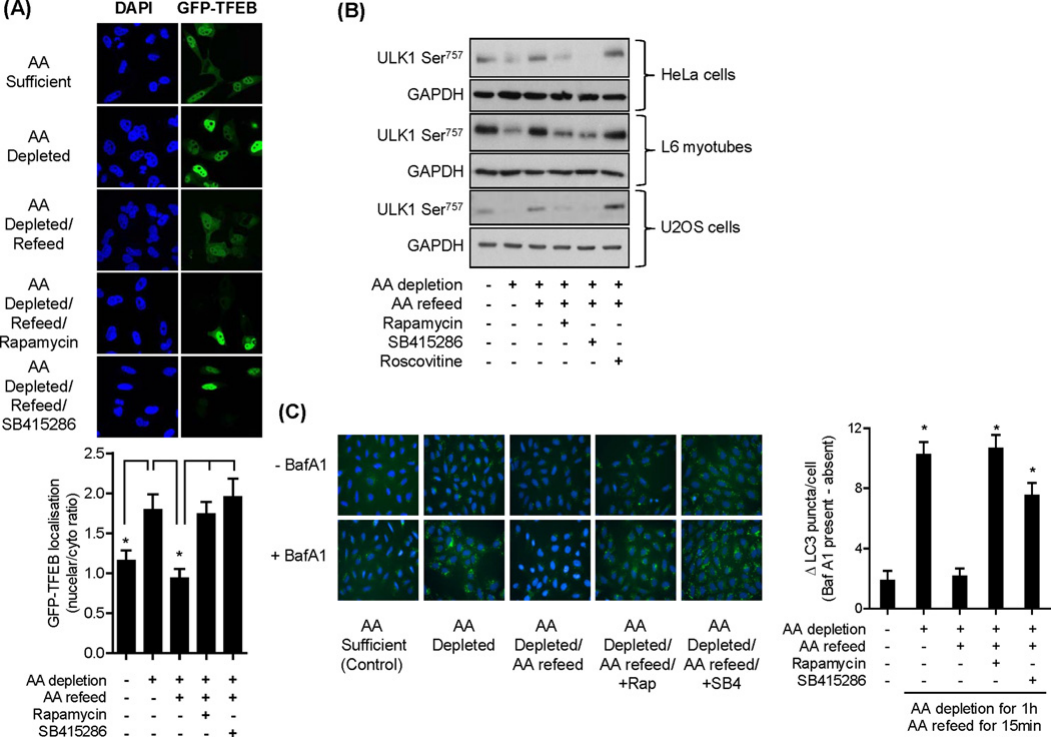
Effects of AA depletion/repletion and GSK3 inhibition upon markers of lysosomal biogenesis and autophagy (**A**) HeLa cells were cultured on coverslips and transfected with GFP-tagged TFEB. Twenty-four hours post transfection, cells were incubated in EBSS and EBSS+AA in the absence or presence of 100 nM rapamycin or 50 μM SB415286 as indicated and cells were fixed, imaged and analysed as described in the Materials and methods section. Asterisks indicate significant (*P*<0.05) changes between indicated bars. (**B**) HeLa cells, L6 myotubes and U2OS cells were subjected to incubation with EBSS containing or lacking AAs for 1 h or alternatively having been depleted of AAs incubated in EBSS containing a 1× AA mix (refeed) for 15 min in the absence/presence of 100 nM rapamycin, 50 μM SB415286 or 30 μM roscovitine as indicated. Cells were lysed and 30 μg of lysate protein was analysed by immunoblotting. (**C**) U20S cells were incubated as in (**B**) but in the absence or presence of 50 nM bafilomycin A1. Cells were fixed and nuclei (blue DAPI) and LC3 puncta (green fluorescence) visualized as described in the Materials and methods section. Asterisks indicate a significant difference between the indicated bars (**A**) or indicate a significant difference in LC3 puncta observed relative to the untreated AA-sufficient control (*P*<0.05).

In addition to p70S6K1 and 4E-BP1, mTORC1 can also phosphorylate the ULK1 protein kinase complex and TFEB, which are respectively involved in the formation of autophagosomes and expression of autophagy and lysosomal genes [34–37]. During AA sufficiency, mTORC1 phosphorylates both ULK1 (thereby suppressing autophagsome formation) and TFEB (which promotes cytosolic retention and thus reduced expression of lysosomal genes). In contrast, mTORC1 inhibition stimulates autophagy and nuclear localization of TFEB as a consequence of its reduced phosphorylation. Consistent with this *modus operandi*, we found that when HeLa cells were subject to AA depletion, there was a modest increase in nuclear localization of GFP-labelled TFEB, whereas refeeding cells with AAs after the depletion period led to significantly greater retention of the transcription factor to the cytosol (Figure 3A). This AA-induced cytosolic retention of TFEB was not apparent in cells treated with either rapamycin or SB415286 during the AA-repletion phase, indicative of mTORC1 inhibition (Figure 3A).

Under conditions of nutrient sufficiency, phosphorylation of ULK1^Ser757^ by mTORC1 is important for inhibiting autophagy induction [38]. Phosphorylated ULK1^Ser757^ was detected in HeLa cells, L6 myotubes as well as U2OS cells (a human osteosarcoma cell line) when incubated in AA-containing buffer. A marked loss in ULK1^Ser757^ phosphorylation was observed when cells were AA-depleted, but this was rapidly reversed upon cellular AA repletion. This AA-induced rephosphorylation of ULK1^Ser757^ was blunted if either rapamycin or SB415286 (but not roscovitine) were present in the AA-repletion buffer (Figure 3B). To assess whether autophagy was induced under these latter circumstances, we monitored the formation of autophagosomes in U2OS cells by visualizing LC3 puncta by immunofluorescence in the absence or presence of the lysosomal proton pump inhibitor, or bafilomycin A1. The inhibitor facilitates an increase in lysosomal pH that helps suppress degradation of autolysosome content, permitting accumulation of LC3-positive autophagosomes as evidence of efficient autophagic flux. Figure 3(C) shows a significant increase in LC3 puncta formation (i.e. autophagy, depicted as green staining) when U2OS cells were subject to AA depletion, which was repressed upon AA resupply following nutrient deprivation. This repression was strongly attenuated under circumstances when mTORC1 signalling was suppressed either directly by rapamycin or via inhibition of GSK3 using SB415286 (Figure 3C).

**Figure 4:**
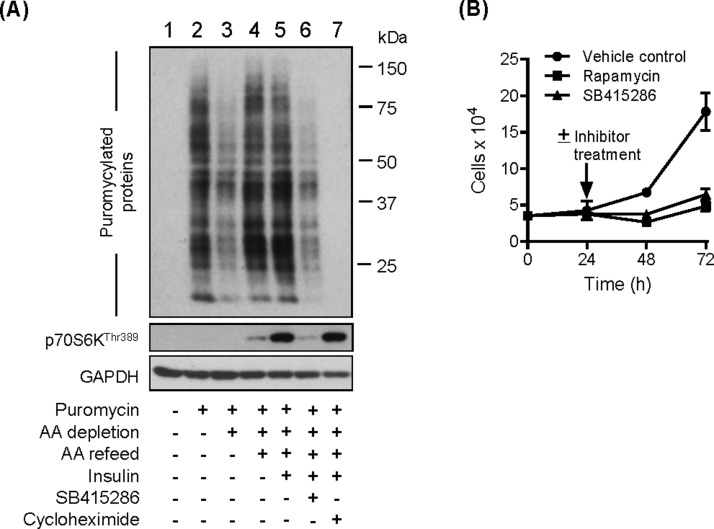
Effect of SB415286 on protein synthesis and cell growth (**A**) HeLa cells were incubated in EBSS containing or lacking AA for 3 h or alternatively having been depleted of AAs incubated in EBSS containing 1× AA mix (refeed) ± 100 nM insulin for 30 min. Cells were incubated with 50 μM SB415286 or 50 μg/ml cycloheximide during the AA depletion and refeed period. Puromycin (1 μM) was added 30 min prior to cell harvest. Cells were lysed and 30 μg of protein was analysed by immunoblotting using antibodies against puromycin or the proteins indicated. (**B**) HEK293T cells were seeded at a density of 3.5 × 10^4^ and treated 24 h post-seeding with vehicle solution and either 100 nM rapamycin or 50 μM SB415286 for up to 72 h. Cell number was quantified as described in the Materials and methods section.

Since mTORC1 positively influences protein synthesis, we explored whether this anabolic process would be affected in cells treated with SB415286. We utilized an anti-puromycin antibody to measure the incorporation of puromycin, a tyrosine-tRNA mimetic, into newly synthesized proteins as readout for this process. As expected, we observed no labelling of proteins in the absence of puromycin (Figure 4A, lane 1) or when cells were incubated with cycloheximide, which would effectively preclude incorporation of puromycin into nascent proteins through its ability to inhibit translocation of mRNA on 80S ribosomes (lane 7). Relative to cells held in AA-containing medium (lane 2) the abundance of puromycylated proteins in AA-deprived cells (lane 3) was reduced consistent with a reduction in protein synthesis that might be expected under nutrient-starved conditions. AA resupply in the absence or presence of insulin (lanes 4 and 5) led to a notable increase in puromycylated proteins that was clearly suppressed if cells had been pre-treated with SB415286 (lane 6). In line with this latter finding, the effects of sustained cell incubation with SB415286 (or rapamycin) were also found to reduce cell proliferation by ∼60% compared with vehicle-treated cells (Figure 4B). This latter finding is in keeping with very recent work showing that four different GSK3 inhibitors (SB216763, AR-A01148, CT99012 and L803-mts) also suppress, by a similar magnitude, the proliferative capacity of MCF-7 cells [19].

### GSK3 inhibition does not affect lysosomal mTOR localization or modify expression of core mTORC1 signalling proteins

**Figure 5:**
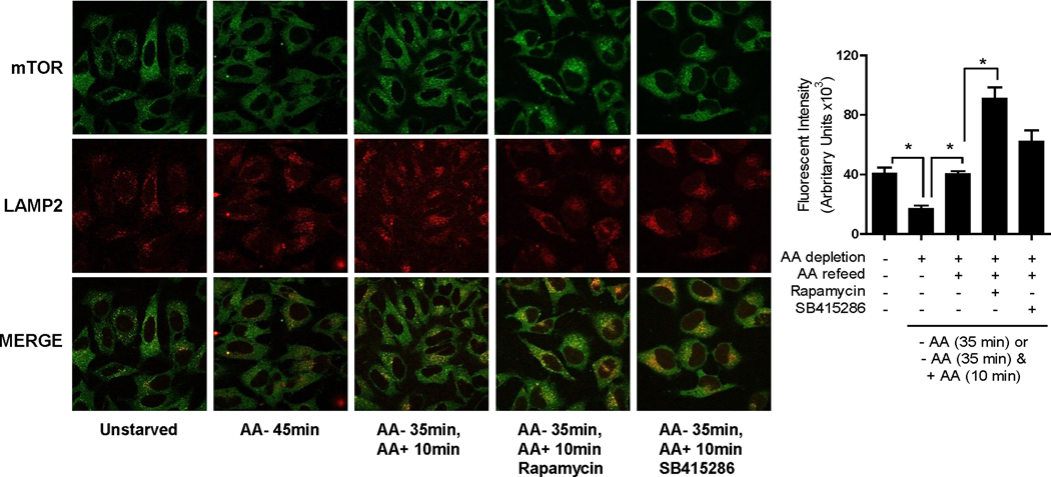
Effect of SB415286 and rapamycin on mTOR localization HeLa cells were cultured in EBSS containing or lacking AA or alternatively having been depleted of AAs incubated in EBSS containing a 1× AA mix (refeed) for the times indicated. In some cases, cells were incubated with 100 nM rapamycin or 50 μM SB415286 for 15 min prior to and for 10 min during the AA-refeed period as indicated. Cells were fixed, and fluorescent labelling and quantification of mTOR co-localization with LAMP2 was carried out as described in the Materials and methods section. Image quantification (means ± S.E.M.) is based on data from at least ten different fields of view. Asterisks indicate significant (*P*<0.05) differences between indicated bars.

Having established that pharmacological inhibition of GSK3 or cellular shRNA-induced depletion of GSK3α and GSK3β results in reduced nutrient-dependent mTORC1 signalling, we attempted to elucidate the basis on which the latter was dependent on GSK3. AA deprivation has been reported to release mTOR from lysosomal membranes, whereas nutrient resupply induces lysosomal re-engagement and activation of the mTORC1 complex [6,39]. To test whether loss of mTORC1 signalling as a consequence of GSK3 inhibition was associated with a disruption in lysosomal mTOR targeting, we compared mTOR localization with that of LAMP2 (a lysosomal protein marker) by immunofluorescence in HeLa cells. Figure 5 shows that, in line with findings from other groups [6], AA depletion caused a net reduction in lysosomal mTOR localization that was restored upon AA refeeding of cells. Intriguingly, whereas both rapamycin and SB415286 suppress nutrient-dependent mTORC1 signalling, this does not involve reduced lysosomal targeting of mTOR which, unexpectedly, we found to be enhanced by both inhibitors during cellular AA repletion. The reasons for this increase in lysosomal mTOR are unclear, but may form part of a feedback mechanism attempting to enhance mTORC1 signalling at the lysosome in response to a reduction in phosphorylation of downstream mTORC1 targets.

**Figure 6:**
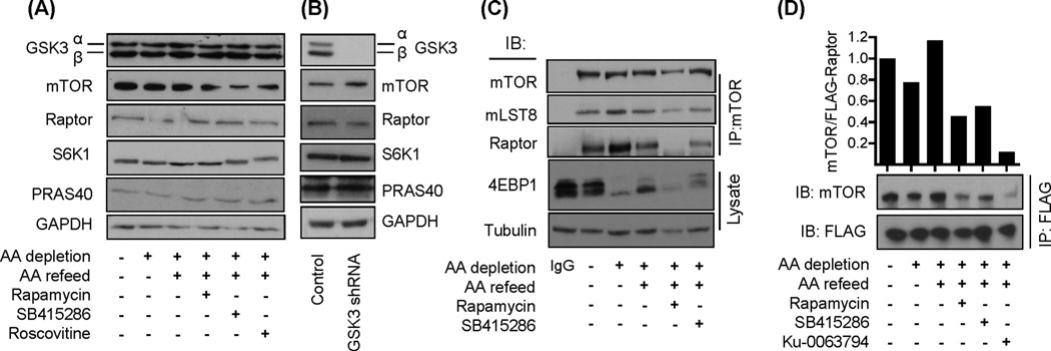
Effects of GSK3 inhibition or shRNA-mediated GSK3α/β silencing on expression of proteins involved in mTOR signalling (**A** and **B**) L6 myotubes were either held in EBSS containing or lacking AAs for 1 h or alternatively, having been AA-depleted, incubated in EBSS containing a 1× AA mix (refeed) for 15 min. Cells were incubated with 100 nM rapamycin, 50 μM SB415286 or 30 μM roscovitine for 15 min prior to and for 15 min during the AA-refeed period as indicated prior to cell lysis. Alternatively, L6 myotubes stably expressing non-specific shRNA or shRNAs targeting GSK3α/GSK3β were lysed for analysis. A 30 μg amount of lysate was analysed by immunoblotting using the antibodies indicated. (**C**) Effect of GSK3 inhibition on raptor and mLST8 association with mTOR and 4E-BP1 phosphorylation. HEK293T cells were incubated in EBSS lacking or containing AAs and inhibitors as in (**A**). Cells were lysed, the lysate was used to immunoprecipitate mTOR, and immunoprecipitates were analysed for proteins indicated. Alternatively, 30 μg of lysate was used for SDS/PAGE and immunoblot analysis of 4E-BP1 or tubulin (gel loading control). (**D**) HEK293T cells overexpressing FLAG–raptor were incubated with EBSS ± AAs as described in (**A**) above and with 100 nM rapamycin, 50 μM SB415286 or 1 μM Ku-0063794, as indicated. Cells were lysed and raptor was immunoprecipitated using anti-FLAG antibodies prior to immunoblotting with anti-mTOR and anti-FLAG antibodies.

Figures 6(A) and 6(B) show that the reduction in mTORC1 signalling observed in cells treated with SB415286 (Figure 1) or GSK3 shRNA (Figure 2C) could not be attributed to gross changes in the expression of mTOR, raptor or PRAS40. Previous work has indicated that raptor dissociation from mTOR contributes to the mechanism by which rapamycin induces inhibition of mTOR function [40]. Immunoprecipitation of mTOR from HEK293T cells led to co-precipitation of raptor and mLST8 which was unaffected by the AA status of the incubation buffer (Figure 6C). However, subjecting cells to incubation with rapamycin led to a noticeable reduction in raptor co-precipitation, indicating a weakening of the mTOR–raptor interaction [41]. We also detected reduced raptor–mTOR association following cell treatment with SB415286 although less robustly than that seen in cells treated with rapamycin. Rapamycin, Ku-0063794 (which inhibits both mTORC1 and mTORC2 [42]) and SB415286 also reduced mTOR–raptor interaction when assessed in FLAG immunoprecipitates from HEK293T cells expressing wt FLAG–raptor (Figure 6D).

### GSK3 mediates raptor phosphorylation on Ser^859^ and influences raptor–mTOR association

**Figure 7:**
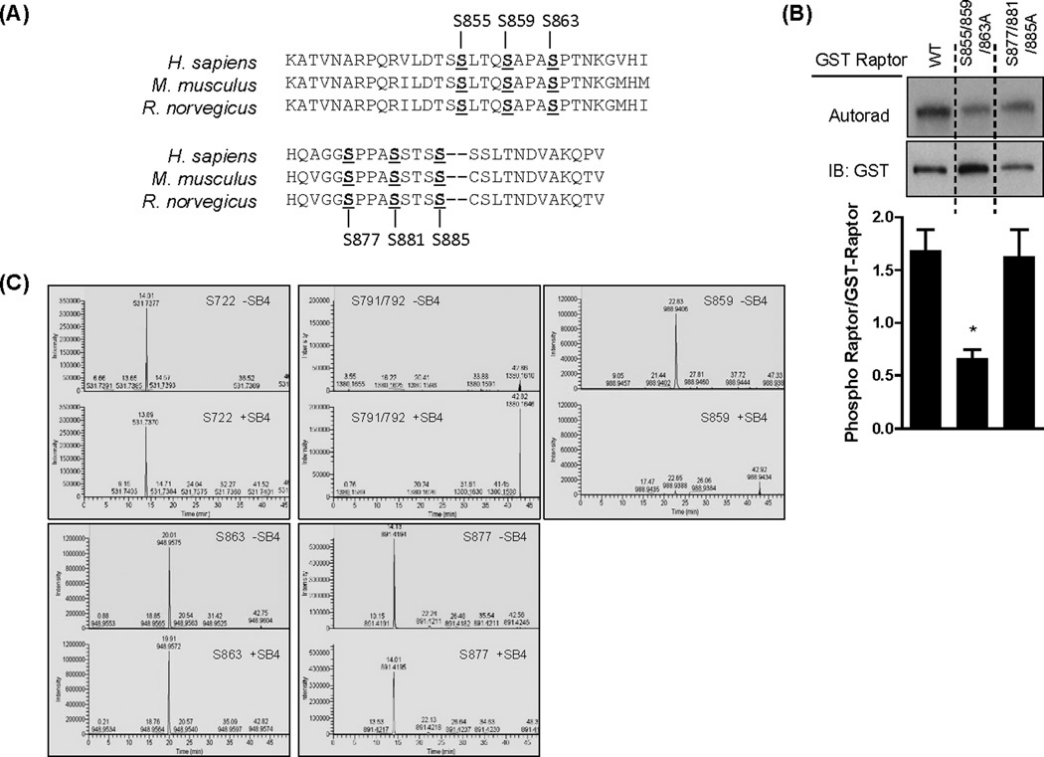
Identification of GSK3-mediated raptor phosphorylation sites (**A**) Alignment of AA sequence of raptor in *Homo sapiens*, *Mus musculus* and *Rattus norvegicus* showing conserved putative GSK3 serine phosphorylation sites. (**B**) HEK293T cells over expressing either GST-tagged wt raptor or GST-tagged mutant raptor were lysed, and GST-tagged raptor was immunoprecipitated. Phosphorylation of the tagged raptor proteins by GSK3β was analysed as described in the Materials and methods section. Analysis of gel loading was confirmed by subsequent blotting of the membrane using anti-GST antibody. *P<0.05, significant difference in the ratio of phosphorylation to total GST–raptor compared with wt raptor. (**C**) HEK293T cells were incubated for 1 h in EBSS containing AAs in the absence or presence of 50 μM SB415286. Cells were lysed and phosphorylation of raptor was analysed by MS, as described in the Materials and methods section.

mTOR signalling can be regulated by stimulus-induced phosphorylation of raptor as seen, for example, in response to hormonal, nutritional and osmotic cues [15,43,44]. To assess whether raptor was a potential GSK3 target, we initially performed *in silico* analysis for putative GSK3 phosphorylation motifs on raptor. Our motif analysis revealed the presence of two putative GSK3 cluster sites (cluster 1: Ser^855^, Ser^859^, Ser^863^ and cluster 2: Ser^877^, Ser^881^, Ser^885^) that map to its central region and which are conserved in human, mouse and rat raptor homologues (Figure 7A). We subsequently expressed wt GST-tagged raptor or raptor constructs in which the serine residues in each identified cluster had been mutated to alanine in HEK293T cells. Expressed GST–proteins were then immunoprecipitated and used as substrates in a GSK3β kinase *in vitro* assay. Figure 7(B) shows that GSK3β-mediated phosphorylation of raptor was reduced significantly (>50%) when serine residues within the first, but not second, cluster were changed to alanine. To identify whether any of the raptor residues identified by *in silico* analysis were *bona fide* GSK3 phosphorylation sites, we performed LC–MS/MS analysis of raptor immunoprecipitated from HEK293T cells incubated in medium containing or lacking SB415286. Our analysis yielded 58% and 60% sequencing coverage in the untreated control and SB415286 samples respectively with all phosphosites localizing to the correct location on the peptides with a greater than 90% probability. Figure 7(C) shows that of the phospho-sites identified by LC–MS/MS analysis, three of these, Ser^859^, Ser^863^ and Ser^877^, are located within the two clusters identified by motif analysis. Ser^855^ in cluster 1 and Ser^881^ and Ser^885^ in cluster 2 identified as putative GSK3 phosphorylation sites were not identified by LC–MS/MS as sites phosphorylated on raptor in our cells. Two additional phospho-sites out with these clusters, Ser^791/792^ and Ser^722^, were also detected. Of the residues detected by LC–MS/MS, only phosphorylation of Ser^859^ was lost following cell treatment with SB415286 (Figure 7C).

**Figure 8:**
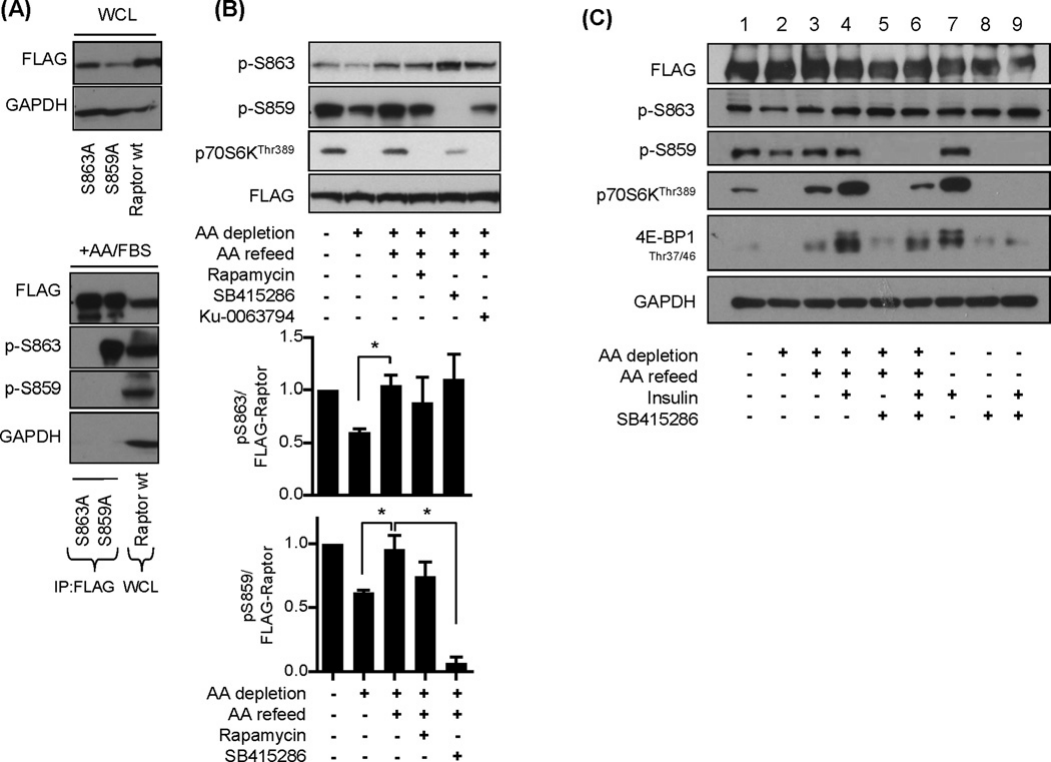
Effects of AA and insulin on raptor and p70S6K1 phosphorylation (**A**) HEK293T cells overexpressing FLAG–raptor, FLAG–raptor S859a or FLAG–raptor S863A mutant were lysed and FLAG-conjugated proteins were immunoprecipitated using anti-FLAG antibodies. Immunoprecipitates and 30 μg of lysate respectively were analysed by immunoblotting for the proteins indicated. (**B**) HEK293T cells overexpressing FLAG–raptor were incubated in EBSS containing or lacking AA for 1 h or alternatively having been depleted of AAs incubated in EBSS containing 1× AA mix (refeed) for 15 min. Cells were incubated with 100 nM rapamycin, 50 μM SB415286 or 1 μM Ku-0063794 for 15 min prior to and for 15 min during the AA-refeed period where indicated. Cells were harvested and 30 μg of protein was analysed by immunoblotting using the antibodies indicated. The lower panel represents quantification of Ser^863^ and Ser^859^ phosphorylation from at least three separate experiments (means ± S.E.M.) (**C**) HEK293 cells overexpressing FLAG–raptor were incubated in EBSS containing or lacking AA for 1 h ± 100 nM insulin (15 min) or alternatively having been depleted of AAs incubated in EBSS containing 1× AA mix (refeed) for 15 min ± 100 nM insulin. Cells were incubated with 50 μM SB415286 for 15 min prior to and for 15 min during the AA refeed period where indicated. Cells were harvested and 30 μg of protein was analysed by immunoblotting using the antibodies indicated.

Phosphorylation of substrates by GSK3 is unusual in that the kinase has a strong preference for substrates that have already undergone a priming phosphorylation event at residue 4 or 5 AAs C-terminal to the GSK3 target site [45]. Based on the finding that Ser^863^ was identified as a phosphorylated residue and is located four residues C-terminal to Ser^859^, we postulated that Ser^863^ may serve as the priming site. To test this proposition we expressed FLAG-tagged wt raptor and FLAG-tagged raptor mutant (S863A and S859A) constructs in HEK293T cells (Figure 8A). The FLAG–raptor proteins were immunoprecipitated using anti-FLAG antibodies and the precipitates screened using phospho-specific antibodies against Ser^863^ and Ser^859^. Figure 8(A) (lower panel) shows that we were unable to detect phosphorylation of either Ser^863^ or Ser^859^ in FLAG–raptor S863A immunoprecipitates, whereas only phosphorylation of Ser^863^ was observed in FLAG–raptor S859A immunoprecipitates. Phosphorylation of both sites was detected in lysates from cells transfected with wt raptor. These findings are consistent with the idea that phosphorylation of Ser^863^ most probably serves to prime raptor phosphorylation of Ser^859^ by GSK3.

Previous studies have reported that, when activated, mTOR mediates phosphorylation of raptor on Ser^863^ [15,43]. To test whether mTOR acts as the priming kinase for Ser^859^ phosphorylation, we immunoprecipitated FLAG–wt raptor from HEK293T cells subjected to AA depletion/resupply in the absence and presence of mTOR and GSK3 inhibitors. We observed a reduction in both Ser^863^ and Ser^859^ phosphorylation when cells were AA depleted for 60 min which was associated with an attendant reduction in p70S6K^Thr389^ phosphorylation (Figure 8B, upper and lower panels). The loss in phosphorylation of both raptor sites and that of p70S6K1^Thr389^ was reversed upon acute AA refeeding of cells. Surprisingly, although p70S6K1^Thr389^ phosphorylation was strongly suppressed by treatment of cells with rapamycin and Ku-0063794 during the AA-repletion phase, neither mTOR inhibitor prevented the AA-induced increase in Ser^863^ and Ser^859^ phosphorylation. In contrast, SB415286 blocked phosphorylation of Ser^859^ without causing any detectable loss of Ser^863^ phosphorylation and also diminished phosphorylation/activation of p70S6K1 (Figure 8B). These observations imply that phosphorylation of Ser^863^ and Ser^859^ can be regulated in an AA-dependent manner and that, although neither serves as an mTOR target site, phosphorylation of Ser^859^ may be necessary for mTORC1-directed signalling.

Since insulin also positively regulates mTORC1 signalling via the Akt–TSC2–Rheb axis, we assessed whether phosphorylation of Ser^863^ and Ser^859^ was modulated by insulin and, if so, whether this was also sensitive to SB415286. Figure 8(C) shows that, although insulin causes a robust increase in p70S6K^Thr389^ phosphorylation, the hormone has no detectable effect on the phosphorylation of Ser^863^ and Ser^859^ in HEK293 cells over-expressing FLAG-tagged wt raptor (compare lanes 1 and 7). Irrespective of whether insulin was present or not, phosphorylation of Ser^859^ (but not Ser^863^) was reduced substantially by the presence of SB415286 and, under these circumstances, we also observed an attendant reduction in p70S6K^Thr389^ and 4E-BP1 phosphorylation (compare lanes 5 and 6 as well as lanes 8 and 9).

**Figure 9:**
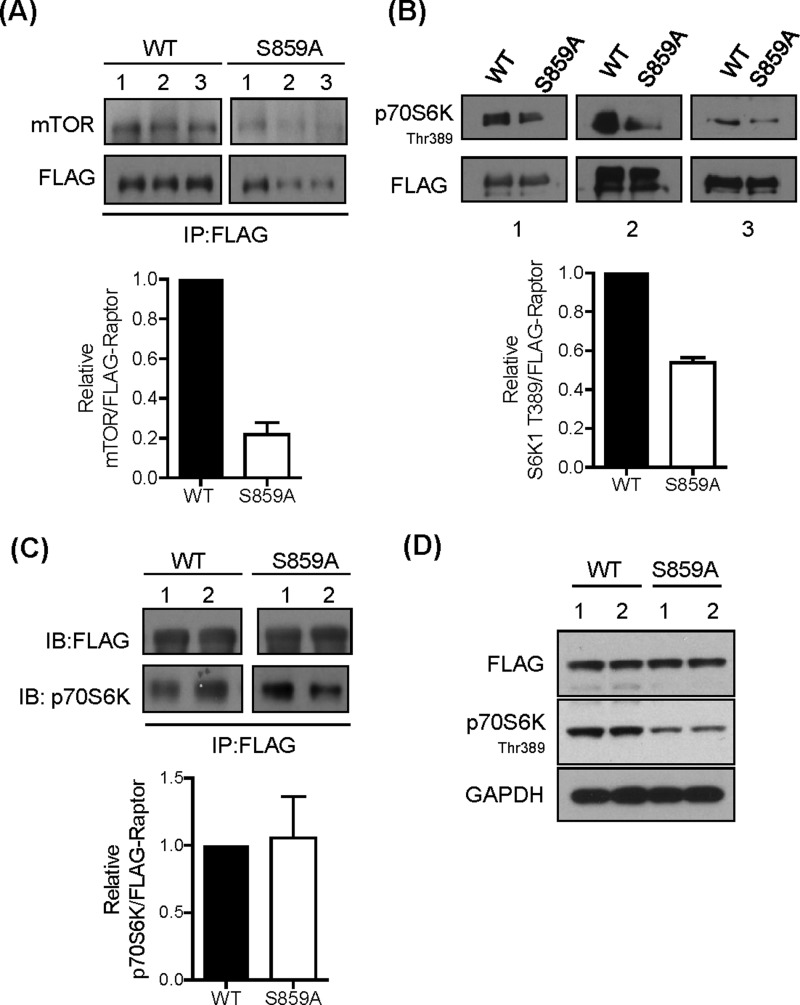
Raptor–mTOR–S6K1 association and mTORC1 signalling (**A**) HEK293T cells overexpressing FLAG–raptor or FLAG–raptor S859A were lysed and FLAG-tagged raptor variants were immunoprecipitated using anti-FLAG antibodies. Immunoprecipitates were analysed by immunoblotting using antibodies against mTOR and FLAG. Three separate experiments were performed. The histogram shows quantification of mTOR co-immunoprecipitated with wt and S859A mutant FLAG–raptor. (**B**) HEK293T cells overexpressing FLAG–raptor or FLAG–raptor S859A mutant were lysed and raptor was immunoprecipitated using anti-FLAG antibody. Raptor-associated mTOR kinase activity was assayed as described in the Materials and methods section using kinase-inactive recombinant p70S6K1 as substrate followed by immunoblotting using the antibody against phospho-70S6K1. The histogram shows quantification of p70S6K phosphorylation in relation to immunoprecipitated wt and S859A mutant FLAG–raptor. (**C**) HEK293T cells overexpressing FLAG–raptor or FLAG–raptor S859A were incubated for 1 h in EBSS containing 1× AA mix. Cells were lysed and FLAG-tagged raptor variants were immunoprecipitated using anti-FLAG antibody. Immunoprecipitates were analysed by immunoblotting for the proteins indicated. The histogram shows quantification of p70S6K that is co-immunoprecipitated with wt and S859A mutant FLAG–raptor from three independent experiments. (**D**) HEK293T cells overexpressing FLAG–raptor or FLAG–raptor S859A mutant were lysed and 30 μg of protein was analysed by immunoblotting for the proteins indicated.

The data presented in Figures 6(C) and 6(D) support the possibility that GSK3 inhibition and raptor phosphorylation of Ser^859^ may influence mTORC1 signalling by modulating the interaction between raptor and mTOR. To test this possibility further, we expressed FLAG-tagged constructs of wt raptor and S859A in HEK293T cells and assessed mTOR activity in FLAG immunoprecipitates. Figure 9(A) shows that the abundance of mTOR was noticeably reduced in FLAG–Raptor S859A precipitates compared with that in wt raptor precipitates. Subsequent analysis of mTOR activity associated with the FLAG immunoprecipitates (using recombinant kinase-dead p70S6K as substrate) demonstrated a near 50% reduction in mTOR activity in FLAG–Raptor S859A precipitates (Figure 9B), consistent with the reduced amount of mTOR associated with the mutated raptor protein (Figure 9A). In contrast, we observed no detectable difference in association of p70S6K1 with wt raptor or the S859A raptor (Figure 9C). This latter observation is entirely consistent with previous work showing that raptor S859A and S863A mutants retain normal capacity for interaction with 4E-BP1, but that mTOR activity associated with the S863A mutant is severely blunted [43]. Compared with HEK293T cells expressing FLAG–wt raptor, those transfected with Flag–Raptor S859A exhibit reduced p70S6K1 phosphorylation (Figure 9D). This finding is in line with the idea that, although the S859A raptor mutant has reduced capacity for interaction with mTOR, it would compete with native raptor for association with mTORC1 and raptor-bound substrates and thereby reduce the pool of these substrates available for mTOR-catalysed phosphorylation.

## DISCUSSION

Hitherto, the idea that GSK3 may influence mTORC1 signalling has primarily been based on evidence indicating an indirect regulatory input that the kinase has upon this key nutrient signalling axis. The co-ordinated phosphorylation and activation of TSC2 by AMPK and GSK3, for example, has been reported to negatively regulate mTORC1 signalling [46]. However, there is also evidence showing that GSK3 promotes S6K1 activation in different cell lines by phosphorylating the kinase on Ser^371^ within its turn motif, which subsequently enhances Thr^389^ phosphorylation by mTOR [47]. It is unclear whether these contrasting observations stem from differences in cell type or cellular context, but recent studies in MCF-7 breast cancer cells demonstrate that GSK3 positively enhances mTORC1 activity and that this stimulatory effect correlates with increased cell growth and proliferation [19]. Although this latter study did not reveal how GSK3 enhanced mTORC1 activity, our observation that GSK3 inhibition suppresses phosphorylation of numerous mTORC1 substrates in multiple cell lines provides yet further support for the view that GSK3 generally conveys a net positive input into the regulation of mTORC1 activity. Furthermore, our subsequent investigation reveals for the first time that it does so by acting directly upon raptor.

Although considerable progress has recently been made in identifying the many proteins involved in signalling AA sufficiency to mTORC1, there is growing appreciation that modulation of raptor phosphorylation on specific sites may serve an important ‘gate-keeping’ function that either promotes or inhibits mTORC1 signalling. Raptor possesses a unique conserved N-terminal half known as the RNC (raptor N-terminal *c*onserved) domain that is followed by a central region containing three HEAT domains and then seven WD40 repeats near the C-terminus [48]. In response to nutrient/energy deprivation for example, AMPK activation results in direct phosphorylation of raptor on residues (Ser^722^ and Ser^792^) located within its central region. Phosphorylation of these sites leads to raptor association with 14-3-3 protein and consequential loss of mTORC1 signalling that, in turn, induces cell cycle arrest [17]. In contrast, phosphorylation of raptor on other serine sites within the central region is considered to be important for promoting mTOR signalling in response to mitogenic and oncogenic activation of the Ras–MAPK pathway [16,49] and for facilitating cell cycle G_2_/M transition when raptor is phosphorylated by cdc2 [18,20]. Our findings demonstrate, for the first time, that raptor is also subject to phosphorylation on Ser^859^ by GSK3 and that phosphorylation of this site supports AA-dependent phosphorylation of multiple mTORC1 targets. In line with previous work [15,43], we find that phosphorylation of Ser^859^ is critically dependent upon phosphorylation of Ser^863^, which we believe primes raptor for phosphorylation on Ser^859^ by GSK3. Since GSK3 is normally active in cells [50] and we find no evidence of it being regulated by changes in AA availability (Supplementary Figure S2), the AA-dependent phosphorylation of Ser^859^ most probably tracks that of Ser^863^ by an AA-regulated kinase activity. Although evidence exists in the literature showing that mTOR can mediate phosphorylation of Ser^863^ [15,43], the observation that neither rapamycin nor Ku-0063794 (mTOR inhibitors) suppress phosphorylation of Ser^863^ in our hands implies that this site is targeted by another, as yet unknown, kinase(s) whose activity may be regulated in an AA-dependent manner. The finding that enhanced raptor phosphorylation of Ser^863^ in response to osmotic stress in HEK293 cells [44] or in contracting skeletal muscle [51] is insensitive to rapamycin strongly indicates the presence of a regulatable Ser^863^ kinase. Alternatively, the enhanced phosphorylation status of Ser^863^ in response to nutrient provision/osmotic stress may be critically dependent upon the inactivation of a phosphatase that normally targets this residue. Understanding how Ser^863^ phosphorylation may be modulated (i.e. whether it involves an AA-regulated priming kinase or phosphatase) represents an important investigative goal of future work if we are to fully understand how AAs enhance mTORC1 signalling.

The notion that GSK3 activity is required for supporting mTORC1-directed nutrient signalling may seem counter-intitutive, given that insulin promotes mTORC1 activation via the Akt–TSC2–Rheb axis whilst inducing phosphorylation and inhibition of GSK3 by an Akt-dependent mechanism. This apparent paradox may be explained on the basis that either (i) insulin targets a discrete subcellular pool of GSK3 that is not directly linked to regulation of the mTORC1 complex, and/or (ii) the extent to which the hormone inhibits GSK3 is insufficient to impact upon mTOR activity. Insulin only causes a partial inactivation (∼30%) of GSK3 (Supplementary Figure S2), which is substantially less than the 80–90% inhibition seen in cells treated with SB415286 [52] or those subjected to shRNA-mediated GSK3 depletion (Figure 2; Supplementary Figure S2) in which we observe a major reduction in mTORC1 signalling to an extent that mimics a state of nutrient insufficiency. This results in suppressed protein synthesis and reduced phosphorylation of substrates such as TFEB and ULK1 that promote a concomitant increase in lysosomal/autophagosomal activity. This is illustrated by our findings that GSK3 inhibition induces greater nuclear localization of TFEB and enhances LC3 puncta formation and is fully in keeping with other recent studies demonstrating that inhibition of GSK3 stimulates lysosomal biogenesis by inducing dissociation of TFEB from 14-3-3 protein and promoting its nuclear localization [53,54]. Although Marchand et al. [54] suggest that GSK3 and mTOR inhibition impinge independently upon TFEB, inhibition of GSK3-mediated mTORC1 activity has recently been shown by others to reduce cell proliferation, increase lysosomal acidification and enhance autophagy in MCF-7 human breast cancer cells [19].

Although previous work has demonstrated that a raptor S863A mutant (in which there would also be an attendant loss of Ser^859^ phosphorylation) is still able to interact with mTOR [15,43], our data indicate that GSK3 inhibition or expression of a S859A mutant significantly reduces raptor–mTOR association. The affinity with which these two proteins interact is known to be modulated in response to insulin and nutrient availability [41] and so it is tempting to speculate that GSK3-mediated phosphorylation of Ser^859^ may induce conformational changes in mTORC1 that permit more effective engagement and phosphorylation of raptor-bound mTOR substrates.

**Figure 10:**
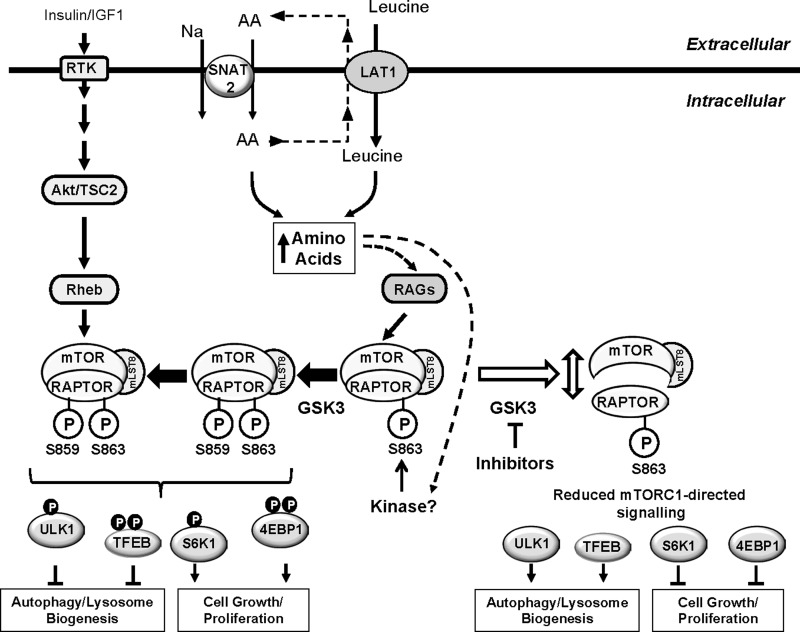
Scheme showing GSK3 involvement in the regulation of mTORC1-directed signalling AA provision regulates RAG-mediated recruitment of the mTORC1 complex to the lysosomal membrane, but also induces phosphorylation of the mTOR scaffold protein raptor on Ser^863^ by an unknown kinase. Phosphorylation of Ser^863^ primes phosphorylation of Ser^859^ by GSK3, which supports mTOR–raptor interaction and mTOR-catalysed phosphorylation of multiple downstream targets such as ULK1 and TFEB (thereby suppressing autophagy/lysosomal biogenesis) and p70S6K1 and 4E-BP1 (which support cell growth and proliferation). GSK3 inhibition leads to a loss in raptor Ser^859^ phosphorylation, reduced mTOR and raptor association and concomitant reduction in mTORC1-directed signalling. Activation of mTORC1 is subject to enhancement by insulin that induces activation of the mTORC1 complex via the Akt–TSC2–Rheb axis. This axis appears not to influence raptor phosphorylation on Ser^863^ or Ser^859^.

In summary, the present study demonstrates that GSK3 activity is required for supporting mTORC1 signalling in response to nutrient availability in a variety of cell types. We propose a model in which GSK3 mediates phosphorylation of raptor on Ser^859^ and suggest that this is crucially dependent upon phosphorylation of Ser^863^ by an, as yet unknown, priming kinase that is likely to be regulated in a nutrient-dependent manner. Phosphorylation of Ser^859^ may stabilize the interaction between mTOR and raptor as reduced phosphorylation of this site promotes their dissociation and a fall in mTORC1-directed signalling (Figure 10). The finding that GSK3 inhibitors suppress mTORC1 signalling and promote lysosomal/autophagic activity thus offers additional pharmacological opportunities for targeting growth/proliferation of cells in which there is heightened mTORC1 activity.

## Abbreviations

4E-BP1: ukaryotic initiation factor 4E-binding protein
p70S6K1: ribosomal S6 kinase 1
AA: amino acid
ACN: acetonitrile
AMPK: AMP-activated protein kinase
DSTT: Division of Signal Transduction Therapy
GAP: GTPase-activating protein
GAPDH: glyceraldehyde-3-phosphate dehydrogenase
GATOR1: GTPase-activating protein towards Rags
GSK3: glycogen synthase kinase 3
HEK: human embryonic kidney
HRP: horseradish peroxidase
IRS: insulin receptor substrate
LAT1: L-type (leucine) amino acid transporter 1
LC3: light chain 3
MLST8: mammalian lethal with SEC13 protein 8
mTOR: mammalian or mechanistic target of rapamycin
mTORC: mTOR complex
PEI: polyethyleneimine
PI3K: phosphoinositide 3-kinase
PRAS40: proline-rich Akt substrate of 40 kDa
ragulator: Rag regulator
raptor: regulatory-associated protein of mTOR
Rheb: Ras homolog enriched in brain
RT: room temperature
SNAT2: sodium-coupled neutral amino acid transporter 2
TFEB: transcription factor EB
TSC: tuberous sclerosis complex
ULK1: uncoordinated-51-like kinase
wt: wild-type
